# Evaluating newly approved drugs for multidrug-resistant tuberculosis (endTB): study protocol for an adaptive, multi-country randomized controlled trial

**DOI:** 10.1186/s13063-021-05491-3

**Published:** 2021-09-25

**Authors:** L. Guglielmetti, E. Ardizzoni, M. Atger, E. Baudin, E. Berikova, M. Bonnet, E. Chang, S. Cloez, J. M. Coit, V. Cox, B. C. de Jong, C. Delifer, J. M. Do, D. Dos Santos Tozzi, V. Ducher, G. Ferlazzo, M. Gouillou, A. Khan, U. Khan, N. Lachenal, A. N. LaHood, L. Lecca, M. Mazmanian, H. McIlleron, M. Moschioni, K. O’Brien, O. Okunbor, L. Oyewusi, S. Panda, S. B. Patil, P. P. J. Phillips, L. Pichon, P. Rupasinghe, M. L. Rich, N. Saluhuddin, K. J. Seung, M. Tamirat, L. Trippa, M. Cellamare, G. E. Velásquez, S. Wasserman, P. J. Zimetbaum, F. Varaine, C. D. Mitnick

**Affiliations:** 1 Médecins Sans Frontières, Paris, France; 2 Sorbonne Université, INSERM, U1135, Centre d’Immunologie Et Des Maladies Infectieuses, Paris, France; 3 Assistance Publique Hôpitaux de Paris, Groupe Hospitalier Universitaire Sorbonne Université, Hôpital Pitié-Salpêtrière, Centre National De Référence Des Mycobactéries Et De La Résistance Des Mycobactéries Aux Antituberculeux, Paris, France; 4 Institute of Tropical Medicine, Antwerp, Belgium; 5 Epicentre, Paris, France; 6 Partners In Health, Astana, Kazakhstan; 7 National Scientific Center of Phthisiopulmonology, Almaty, Kazakhstan; 8 Institut de Recherche pour le Développement/INSERM U1175/UMI233/ Université de Montpellier, Montpellier, France; 9 Médecins Sans Frontières, Toronto, Ontario Canada; 10 Department of Global Health and Social Medicine, Harvard Medical School, Boston, MA USA; 11 Centre for Infectious Disease Epidemiology and Research, School of Public Health and Medicine, Faculty of Health Sciences, University of Cape Town, Cape Town, South Africa; 12 Southern Africa Medical Unit, Médecins Sans Frontières, Cape Town, South Africa; 13 Interactive Research and Development, Karachi, Pakistan; 14 Socios En Salud-Sucursal Peru, Lima, Peru; 15 Assistance Publique Hôpitaux de Paris, Unité de Recherche Clinique, Hôpital Pitié-Salpêtrière, Paris, France; 16 Division of Clinical Pharmacology, Department of Medicine, University of Cape Town, Cape Town, South Africa; 17 Wellcome Centre for Infectious Diseases Research in Africa, Institute of Infectious Disease and Molecular Medicine, University of Cape Town, Cape Town, South Africa; 18 Abiomed, Inc., Danvers, MA USA; 19 Social & Scientific Systems-DLH, Silver Spring, MD USA; 20 Partners In Health, Maseru, Lesotho; 21 Epidemiology and Communicable Diseases Division, Indian Council of Medical Research, Pune, India; 22 Indian Council of Medical Research – National AIDS Research Institute, Pune, India; 23 University of San Francisco Center for Tuberculosis, San Francisco, CA USA; 24 Partners In Health, Boston, MA USA; 25 Division of Global Health Equity, Brigham and Women’s Hospital, Boston, MA USA; 26 Department of Infectious Diseases, Indus Hospital, Karachi, Pakistan; 27 Dana-Farber Cancer Institute, Boston, MA USA; 28 Harvard T.H. Chan School of Public Health, Boston, MA USA; 29 Division of Infectious Diseases, Brigham and Women’s Hospital, Boston, MA USA; 30 Wellcome Centre for Infectious Diseases Research in Africa, Department of Medicine, University of Cape Town, Cape Town, South Africa; 31 Division of Infectious Diseases and HIV Medicine, Department of Medicine, Groote Schuur Hospital and University of Cape Town, Cape Town, South Africa; 32 Harvard Medical School, Boston, MA USA; 33 Beth Israel Deaconess Medical Center, Boston, MA USA

**Keywords:** Rifampin-resistant tuberculosis, Rifampicin-resistant tuberculosis, Bedaquiline, Delamanid, Linezolid, Clofazimine, Fluoroquinolone, Pyrazinamide, Treatment shortening, MDR-TB, Non-inferiority, Bayesian adaptive randomization

## Abstract

**Background:**

Treatment of multidrug- and rifampin-resistant tuberculosis (MDR/RR-TB) is expensive, labour-intensive, and associated with substantial adverse events and poor outcomes. While most MDR/RR-TB patients do not receive treatment, many who do are treated for 18 months or more. A shorter all-oral regimen is currently recommended for only a sub-set of MDR/RR-TB. Its use is only conditionally recommended because of very low-quality evidence underpinning the recommendation. Novel combinations of newer and repurposed drugs bring hope in the fight against MDR/RR-TB, but their use has not been optimized in all-oral, shorter regimens. This has greatly limited their impact on the burden of disease. There is, therefore, dire need for high-quality evidence on the performance of new, shortened, injectable-sparing regimens for MDR-TB which can be adapted to individual patients and different settings.

**Methods:**

endTB is a phase III, pragmatic, multi-country, adaptive, randomized, controlled, parallel, open-label clinical trial evaluating the efficacy and safety of shorter treatment regimens containing new drugs for patients with fluoroquinolone-susceptible, rifampin-resistant tuberculosis. Study participants are randomized to either the control arm, based on the current standard of care for MDR/RR-TB, or to one of five 39-week multi-drug regimens containing newly approved and repurposed drugs. Study participation in all arms lasts at least 73 and up to 104 weeks post-randomization. Randomization is response-adapted using interim Bayesian analysis of efficacy endpoints. The primary objective is to assess whether the efficacy of experimental regimens at 73 weeks is non-inferior to that of the control. A sample size of 750 patients across 6 arms affords at least 80% power to detect the non-inferiority of at least 1 (and up to 3) experimental regimens, with a one-sided alpha of 0.025 and a non-inferiority margin of 12%, against the control in both modified intention-to-treat and per protocol populations.

**Discussion:**

The lack of a safe and effective regimen that can be used in all patients is a major obstacle to delivering appropriate treatment to all patients with active MDR/RR-TB. Identifying multiple shorter, safe, and effective regimens has the potential to greatly reduce the burden of this deadly disease worldwide.

**Trial registration:**

ClinicalTrials.gov Identifier NCT02754765. Registered on 28 April 2016; the record was last updated for study protocol version 3.3, on 27 August 2019.

## Administrative information

Note: the numbers in curly brackets in this protocol refer to SPIRIT checklist item numbers. The order of the items has been modified to group similar items (see http://www.equator-network.org/reporting-guidelines/spirit-2013-statement-defining-standard-protocol-items-for-clinical-trials/).

**Table:** 

Title {1}	Evaluating newly approved drugs for multidrug-resistant tuberculosis (endTB): study protocol for an adaptive, multi-country randomized controlled trial
Trial registration {2a and 2b}.	The endTB trial (evaluating newly approved drugs for multidrug-resistant tuberculosis, clinicaltrials.gov NCT02754765)
Protocol version {3}	The clinicaltrials.gov record was last updated for study protocol version 3.3, on August 27, 2019
Funding {4}	The trial and the preparation of this manuscript are funded by a grant provided by Unitaid. The funding source had no role in the study design; in the collection, management, analysis, or interpretation of data; in the writing of the report; or in the decision to submit the report for publication. GEV received funding from the National Institute of Allergy and Infectious Diseases at the U.S. National Institutes of Health (grant numbers K08 AI141740, L30 AI120170, and P30 AI060354), the Dr. Lynne Reid/Drs. Eleanor and Miles Shore Fellowship at Harvard Medical School, the Burke Global Health Fellowship at the Harvard Global Health Institute, and the Harvard University Center for AIDS Research.
Author details {5a}	^1^ Médecins Sans Frontières, Paris, France. ^2^ Sorbonne Université, INSERM, U1135, Centre d’Immunologie Et Des Maladies Infectieuses, Paris, France. ^3^ Assistance Publique Hôpitaux de Paris (APHP), Groupe Hospitalier Universitaire Sorbonne Université, Hôpital Pitié-Salpêtrière, Centre National De Référence Des Mycobactéries Et De La Résistance Des Mycobactéries Aux Antituberculeux, Paris, France. ^4^ Institute of Tropical Medicine (ITM), Antwerp, Belgium. ^5^ Epicentre, Paris, France. ^6^ Partners In Health, Astana, Kazakhstan. ^7^ National Scientific Center of Phthisiopulmonology, Almaty, Kazakhstan. ^8^ IRD UMI233/ INSERM U1175/Université de Montpellier, Montpellier, France. ^9^ Médecins Sans Frontières, Toronto, Ontario. ^10^ Department of Global Health and Social Medicine, Harvard Medical School, Boston, Massachusetts. ^11^ Centre for Infectious Disease Epidemiology and Research (CIDER), School of Public Health and Medicine, Faculty of Health Sciences, University of Cape Town, South Africa. ^12^ Southern Africa Medical Unit, Médecins Sans Frontières, Cape Town, South Africa. ^13^ Interactive Research and Development, Karachi, Pakistan. ^14^ Socios En Salud-Sucursal Peru, Lima, Peru. ^15^ Assistance Publique Hôpitaux de Paris (APHP), Unité de Recherche Clinique, Hôpital Pitié-Salpêtrière, Paris, France. ^16^ Division of Clinical Pharmacology, Department of Medicine, University of Cape Town, Cape Town, South Africa. ^17^ Wellcome Centre for Infectious Diseases Research in Africa (CIDRI-Africa), Institute of Infectious Disease and Molecular Medicine, University of Cape Town, Cape Town, South Africa. ^18^ Abiomed, Inc. Danvers, Massachusetts. ^19^ Social & Scientific Systems-DLH, Silver Spring, Maryland. ^20^ Partners In Health, Maseru, Lesotho. ^21^ Epidemiology and Communicable Diseases Division, Indian Council of Medical Research, Pune, India. ^22^ Indian Council of Medical Research (ICMR) – National AIDS Research Institute, Pune, India. ^23^ University of San Francisco (UCSF) Center for Tuberculosis, San Francisco, California. ^24^ Partners In Health, Boston, Massachusetts. ^25^ Division of Global Health Equity, Brigham and Women’s Hospital, Boston, Massachusetts. ^26^ Department of Infectious Diseases, Indus Hospital, Karachi, Pakistan. ^27^ Dana-Farber Cancer Institute, Boston, Massachusetts. ^28^ Harvard T.H. Chan School of Public Health, Boston, Massachusetts. ^29^ Division of Infectious Diseases, Brigham and Women’s Hospital, Boston, Massachusetts. ^30^ Wellcome Centre for Infectious Diseases Research in Africa, Department of Medicine, University of Cape Town, Cape Town, South Africa. ^31^ Division of Infectious Diseases and HIV Medicine, Department of Medicine, Groote Schuur Hospital and University of Cape Town, Cape Town, South Africa. ^32^ Harvard Medical School, Boston, Massachusetts. ^33^ Beth Israel Deaconess Medical Center, Boston, Massachusetts.
Name and contact information for the trial sponsor {5b}	Dr. Francis Varaine, Médecins Sans Frontières, Paris, France. francis.varaine@paris.msf.org
Role of sponsor {5c}	The trial is sponsored by Médecins Sans Frontières. The sponsor is involved in all trial activities, including study design, data collection and analysis, and writing/submission of the report. The sponsor has ultimate authority over these activities. The sponsor is responsible for (delegation of) communicating protocol modifications to investigators, participants, and registries after regulatory and ethics approvals. The design and implementation of the trial are supported and overseen by the following external, independent committees: a Data Safety Monitoring Board, a Scientific Advisory Committee, the Global Tuberculosis Community Advisory Board (TB-CAB).

## Background {6a}

The endTB trial (evaluating newly approved drugs for multidrug-resistant tuberculosis, clinicaltrials.gov Identifier NCT02754765) was designed in 2015 to avail of and optimize exciting, new developments in treatment for drug-resistant tuberculosis (DR-TB). That year, there were an estimated 580,000 new cases of TB caused by organisms resistant to the most potent first-line anti-TB drugs (at least rifampin, RR-TB; or at least isoniazid and rifampin, MDR-TB). Only 132,120 cases (22.7%) of MDR/RR-TB were detected and 125,000 (21.5%) received treatment with second-line TB drugs. Roughly half were cured [1]. The consequences of this situation are dire: death is common among MDR/RR-TB patients who experience unfavourable treatment outcomes [2, 3]. Many of those treated receive conventional regimens comprising 18–24 months of toxic, poorly tolerated multidrug treatment [4]. Cure is reported in only 57% globally and loss to follow-up occurs in 16% [5, 6]. Toxicity occurs in nearly all patients receiving conventional treatment for MDR-TB [7]. In two pivotal trials of new anti-TB drugs, patients in the control arms received placebo plus background conventional MDR-TB treatment regimens: approximately 95% experienced adverse events. And serious adverse events were reported in 19% of participants randomized to the control arm in the bedaquiline trial (NCT00449644) and 9% in the delamanid trial (NCT00685360) [8, 9]. Observational cohorts corroborate these findings, with 73 to 79% reported to experience toxicity [10, 11]. This complicates the management of MDR-TB treatment and frequently leads to suspension and replacement of drug(s) in conventional regimens [12, 13]. Toxicity is a major driver of loss to follow-up [14] and is associated with lower rates of culture conversion [15].

Recent developments offered promise of improvement; however, this potential has yet to be fully realized. A shorter alternative regimen with promising efficacy emerged for MDR-TB in patients with limited prior treatment exposure and resistance [16, 17]. The “STREAM” or “Bangladesh” regimen relies on drugs commonly used in first-line treatment and/or in MDR/RR-TB treatment for 20 years; in some settings, resistance to these drugs is common [18, 19]. Uptake of this innovation was limited by this reality and also by the simultaneous emergence of the first new anti-TB drugs in 50 years. In 2012 and 2014, bedaquiline and delamanid, respectively, received conditional approval from stringent regulatory authorities (SRA) for the treatment of MDR-TB. The pivotal trials of these drugs added each to a background regimen, improving interim and long-term outcomes, but retaining the toxic, long, conventional (injectable-containing) regimen [8, 9, 20, 21]. Murine and clinical studies supported the shortening potential of combinations of new agents, co-administered with repurposed drugs [22–31]. Such combinations, however, have not been optimized and examined rigorously through randomized, internally, concurrently controlled clinical trials with participants followed to ascertain relapse-free cure. Nevertheless, there has been substantial pressure to modify guidance in this breach between the unpalatable conventional regimen and rigorously studied shorter, injectable-sparing regimens. Policy changes have, therefore, generally comprised “conditional recommendations” based on “very low-quality” evidence. The endTB trial aims to close this gap by studying in a randomized, internally controlled trial, shorter treatments composed of novel combinations of newer and repurposed drugs. The endTB trial tests experimental regimen combinations drawn from among the following drugs: bedaquiline, delamanid, clofazimine, linezolid, moxifloxacin or levofloxacin, and pyrazinamide. endTB regimen selection was guided by the following principles. Regimens should: contain at least one new drug class, include 3 to 5 likely effective drugs preferentially those without extensive prior use, be effective against a range of MDR/RR strains of* M. tuberculosis*, shorten duration, be all-oral, and have a simple dosing profile, acceptable side effect profile, and minimal drug-drug interactions with antiretrovirals [32]. This phase 3 trial evaluates five novel, 9-month, all oral, treatment regimens for patients with fluoroquinolone-susceptible MDR/RR-TB. The study protocol is presented here.

### Objectives {7}

The primary objective of the endTB trial is to assess whether the efficacy of experimental regimens at 73 weeks post-randomization is non-inferior to that of the control. Secondary objectives include efficacy comparisons at 8, 39, and 104 weeks. Safety objectives include comparisons between the experimental and control arms of the proportion of patients who died or who experience grade 3 or higher adverse events (AEs), serious adverse events (SAEs) of any grade, and adverse events of special interest (AESI), at 73 and 104 weeks. Important exploratory objectives include comparing the effect of two linezolid dose-reduction strategies on toxicity and efficacy of experimental regimens.

## Methods

The study protocol hereby presented contains all items defined by the Standard Protocol Items: Recommendations for Interventional Trials (SPIRIT) statement. A completed SPIRIT checklist is provided (Supplement 1).

### Design {8}

endTB is a phase III, pragmatic, multi-country, adaptive, randomized, controlled, parallel, non-inferiority open-label clinical trial evaluating the efficacy and safety of five 9-month treatment regimens containing recently approved drugs for MDR-TB compared to a control that is the current standard of care for MDR-TB.

#### Randomization, masking, and blinding {16a}{16b}{16c}{17a}

Randomization is web-based (with voice backup), and adapted randomization lists are uploaded to the web-based platform. The study is open label; the regimens are not masked. Microbiology staff who perform testing that is core to efficacy assessments are blinded to treatment assignment. Central investigators are also blinded. Since site investigators are not blinded to assignment and are possibly influenced by opinions about regimen allocation, permanent regimen changes are made with input from the independent Clinical Advisory Committee (CAC), staffed by expert MDR-TB clinicians. The CAC also validates study endpoints that are assigned by local investigators. CAC members do not provide any input on the study protocol and are not involved in the study analysis.

Secondary, balanced (1:1 allocation ratio) randomization to linezolid dose-reduction strategy occurs among patients in the experimental arms at 16 weeks post randomization or earlier if required for toxicity.

#### Interim analysis and stopping {21b}

Randomization is outcome adaptive using interim Bayesian analyses of efficacy endpoints; it has been described previously [33, 34] and is summarized here. The initial randomization list used fixed block sizes with balanced allocation to all 6 arms. After approximately 30 participants are assigned to each arm, the response adaptation begins. At approximately monthly intervals, interim treatment effect (at 8 and 39 weeks) is estimated for each arm, relative to the control, through Bayesian modelling. Randomization probabilities are updated after each interim analysis; higher probability of randomization is assigned to arms with greater interim treatment effect. The probability of assignment to the control arm matches the probability of assignment to the most effective experimental arm. The interim analysis is conducted by a Bayesian statistician (MC) who is not involved in study operations.

Further efficacy review may inform dropping of regimen(s) for futility. Stopping an arm early may be indicated if the posterior probability of a regimen being non-inferior to the control at week 73 falls below a predefined threshold of 5%. Conversely, if the posterior probability is at least 95% that a regimen is superior to the control at week 73, stopping for efficacy will be considered. This may trigger a review by the DSMB to decide if this superior regimen should be stopped (i.e. no more participants are randomized to this regimen) or if the evidence is to be balanced with the safety data.

#### Regimen composition and duration experimental arms {11a}

Experimental arms comprise standardized, four- or five-drug combinations of the following: delamanid, clofazimine, linezolid, pyrazinamide, and a fluoroquinolone (moxifloxacin or levofloxacin); see Tables 1 and 2 for composition and dosing, respectively. Selected regimens combine drugs with distinct mechanisms of action, bactericidal (bedaquiline, delamanid, linezolid, fluoroquinolones) and sterilizing (bedaquiline, clofazimine, delamanid, linezolid, pyrazinamide [35–37]) activity against *Mycobacterium tuberculosis*, and activity in different microenvironments (acid, hypoxic, etc.). Possible overlapping toxicity, particularly QT interval prolongation that could predispose to cardiotoxicity, is managed by including no more than 2 drugs considered to be major QT interval prolongers (bedaquiline, clofazimine, and moxifloxacin) in a regimen. All regimens contain a fluoroquinolone and pyrazinamide, the only drugs to which prior exposure is widespread. The fluoroquinolones, especially the later-generation class members, are a cornerstone of treatment for MDR-TB. In vitro, animal, and human studies all support the inclusion of this drug class for the treatment of resistant TB [38, 39] in patients with isolates susceptible to fluoroquinolones. Although pyrazinamide is linked to toxicity, especially hepatotoxicity, WHO previously recommended inclusion of pyrazinamide in all MDR-TB regimens [40]. Currently, it is recommended to count pyrazinamide as an effective drug only in case drug susceptibility testing confirms susceptibility [4]. Acknowledging the important role of pyrazinamide (when active) in TB treatment shortening [41], and the uncertainty about activity at the time of treatment initiation, all endTB experimental regimens contain pyrazinamide plus at least three other likely effective anti-TB agents. This is consistent with WHO guidance on the treatment of MDR-TB in place when the trial was designed [42].

**Table 1:** Description of endTB treatment arms {11a}

Trial regimens	Bedaquiline	Delamanid	Clofazimine	Linezolid	Fluoroquinolone	Pyrazinamide
**endTB 1**	Bdq			Lzd	Mfx	Z
**endTB 2**	Bdq		Cfz	Lzd	Lfx	Z
**endTB 3**	Bdq	Dlm		Lzd	Lfx	Z
**endTB 4**		Dlm	Cfz	Lzd	Lxf	Z
**endTB 5**		Dlm	Cfz		Mfx	Z
**endTB 6** (**Control)**	Standard of care control, composed according to latest WHO Guidelines, including the possible use of Dlm or Bdq.					

*Abbreviations: Bdq*, bedaquiline; *Dlm*, delamanid; *Cfz*, clofazimine; *Lzd*, linezolid; *Mfx*, moxifloxacin; *Lfx*, levofloxacin; *Z*, pyrazinamide

**Table 2:** Experimental arm drug doses by weight bands {11a}

Drug^**a**^	Weight band (kg)				
	30–35	>35–45	>45–55	>55–70	>70
**Bedaquiline**	400 mg QD x 2 weeks^b^ followed by 200 mg 3x/week				
**Delamanid**	100 mg BID				
**Moxifloxacin**	400 mg				
**Levofloxacin**	750 mg	1000 mg			
**Linezolid**^**c**^	600 mg QD up to week 16 (followed by 300 mg QD or 600 mg 3×/week)				
**Clofazimine**	100 mg				
**Pyrazinamide**	800 mg	1200 mg	1600 mg		2000 mg

^a^Dosing is once a day unless otherwise indicated

^b^The loading dose of bedaquiline treatment comprises 2 weeks of 400 mg QD. If the patient is already receiving bedaquiline treatment, bedaquiline within the experimental regimen should be dosed as a continuation of that treatment (i.e. only remaining doses of the 2-week loading dose should be administered and a load should not be restarted if it was already completed at time of study treatment initiation)

^c^Linezolid dosing is routinely modified at week 16 or sooner if necessary to reduce toxicity related to linezolid. The modification entails either decreased (300 mg daily) or intermittent (600 mg 3×/week) dosing as defined by balanced randomization

Treatment in experimental arms is for 39 weeks; participants in the experimental arms are allowed up to 47 weeks to complete the 39-week treatment course. Of note, while linezolid dosing starts at 600 mg/day, the aforementioned dose-reduction randomization assigns experimental arm patients to receive either linezolid at 300 mg daily or at 600 mg three times/week starting at 16 weeks (or earlier, if indicated by toxicity).

#### Regimen composition and duration control arm {6b}

Control-arm treatment is constructed according to the latest WHO recommendations [4] and local guidance: composition of the regimens may therefore change over the course of the trial. Control-arm treatment may contain one or both recently approved drugs (bedaquiline or delamanid) in addition to companion drugs. Duration is variable: the conventional regimen is delivered for approximately 86 weeks. Shorter regimens that are endorsed by WHO for routine use, are permitted [43]. Oral drugs are delivered 7 days/week in both experimental and control arms. Injectable drugs (used only rarely under protocol version 3.3) in the control arm are delivered at least 6 days/week, according to local practices. All drug intakes are directly observed.

#### Follow-up duration

Study participation is for up to 104 weeks post-randomization; those participants remaining in follow-up when the last participant completes 73 weeks will have their follow-up truncated.

### Setting {9}

The endTB trial is jointly coordinated by members of the endTB consortium: Interactive Research and Development (IRD), Médecins Sans Frontières (MSF), and Partners In Health (PIH) and their research partners, Harvard Medical School, Epicentre, and the Institute of Tropical Medicine of Antwerp (ITM); most coordination is based at MSF. The trial is implemented in countries selected for the following: a significant burden of MDR/RR-TB; the presence of a member institution of the endTB consortium or another entity experienced in TB clinical trials and an existing relationship between TB services and the aforementioned group; clinical trial experience or potential (established through a multi-step site-assessment process); suitable MDR-TB clinical management systems, regulatory environment, research pharmacy capability, and micro/molecular biology services; and heterogeneity in DR-TB patient characteristics (geography, resistance, comorbidities, risk-factor profiles). The list of participating countries and sites is available on ClinicalTrials.gov.

### Study population

#### Inclusion/exclusion {10}

The study population comprises males and females aged ≥15 years with pulmonary TB suspected or confirmed to be resistant to rifampin, without known fluoroquinolone resistance. Inclusion and exclusion criteria are detailed in Table 3. Inclusion criteria include pulmonary TB caused by fluoroquinolone-susceptible and rifampin-resistant *M. tuberculosis*, written informed consent, willingness to use effective contraception, and expected ability to remain locatable in study catchment area for the duration of study participation. Patients are excluded for known allergies or hypersensitivity to any of the investigational drugs; pregnancy or breastfeeding; prior exposure/resistance to bedaquiline, delamanid, linezolid, or clofazimine; any second-line anti-TB drug exposure for 15 days or more immediately prior to the screening visit date; abnormal haematology, biochemistry; cardiac risk factors; required use of a contraindicated medication; social or medical conditions, which, in the opinion of the investigator, would make study participation unsafe. HIV positivity, regardless of CD4 lymphocyte counts, is not a reason for exclusion. Fluoroquinolone resistance on phenotypic test will result in late exclusion.

**Table 3:** Inclusion and exclusion criteria for study.

**Inclusion criteria**	
• Documented pulmonary TB due to strains of *Mycobacterium tuberculosis* resistant to rifampin and susceptible to fluoroquinolones • Diagnosed by validated rapid molecular test • ≥ 15 years of age • Willingness to use effective contraception • Provision of informed consent for study participation • Residence in a dwelling that can be located by study staff and an expectation to remain in the area for the duration of the study	
**Exclusion criteria**	
• Patients with known allergies or hypersensitivity to any of the investigational drugs • Patients known to be pregnant or unwilling or unable to stop breastfeeding an infant • Patients unable to comply with treatment or follow-up schedule • Patients with exposure (intake for 30 days or more) in the past 5 years to bedaquiline, delamanid, linezolid, or clofazimine, or with proven or likely resistance to bedaquiline, delamanid, linezolid, or clofazimine • Patients who have received second-line drugs for 15 days or more prior to the screening visit date in the current MDR-TB treatment episode • Patients with one or more of the following laboratory results: - Grade 3 or higher haemoglobin, calcium, magnesium, creatinine, or bilirubin - Grade 2 or higher potassium, aspartate aminotransferase, alanine aminotransferase, or total bilirubin - Albumin < 2.8 g/dL - Grade 4 result on any other screening laboratory tests • Patients with cardiac risk factors including ECG abnormalities (i.e. QTcF≥450 ms), pacemaker implant, and personal/family history of cardiovascular disease (i.e. long QT syndrome, left or right bundle branch block) • Patients requiring continued use of a contraindicated medication • Patients currently taking part in another trial of medicinal product • Patients with any condition (social or medical) which, in the opinion of the investigator, would make study participation unsafe	

#### Study treatment discontinuation and study withdrawal {11b}{11d}

Study treatment may be discontinued in the following situations: (1) pregnancy or breastfeeding, (2) required use of prohibited concomitant medications, (3) indications of treatment failure, and (4) any other condition (social or medical) which the site principal investigator believes would make study participation unsafe. Prohibited concomitant medications depend on treatment received by the participant. They include moderate and strong CYP3A4 inhibitors and inducers for bedaquiline-containing regimens; strong inducers are also disallowed with delamanid-containing regimens. With linezolid-containing regimens, disallowed medications are any medicinal product that inhibits monoamine oxidases A or B, tricyclic antidepressants, selective serotonin reuptake inhibitors, selective serotonin/norepinephrine reuptake inhibitor, triptans, and other serotoninergic agents. Decisions to permanently discontinue study treatment are made in consultation with the Clinical Advisory Committee. Participants will be referred to local services for treatment. Discontinuation of treatment for the above-specified reasons, and alteration of the assigned study regimen, results in withdrawal from study. Study participation also ends if consent is withdrawn. In case study participation is ended prematurely, an early termination visit will be performed if possible. In addition, participants discontinuing treatment before the week 73 visit will be encouraged to perform post-termination follow-up visits at weeks 39 and 73, as needed.

#### Recruitment and retention {11c}{15}{18b}{26a}{22}{30}

Prospective participants are identified by facility staff in inpatient or outpatient facilities, located in the study catchment areas, that provide TB diagnosis and/or treatment. Patients who agree to be evaluated for the study are referred to study staff. Study staff explain the study, including potential risks and benefits associated with participation. Subsequently, screening consent is obtained from participants (or from parent or guardian, in case of minors, who also provide assent) by site investigators or other delegated site staff prior to any trial-specific evaluation. Baseline consent and randomization follow in those who are eligible.

Retention in the study is ensured through comprehensive, individualized patient support, including adherence enablers and home visits as needed. During treatment, adherence is monitored at every visit and adherence counselling is provided by specialized staff. All transport costs encountered by participants are covered by the study. Food support is provided. Participants requiring care for comorbidities (e.g. HIV, diabetes mellitus) receive all care in the study setting or through facilitated referrals to local providers.

Adverse events are solicited at all study visits; spontaneous reporting of adverse events can also occur at scheduled study visits, through daily treatment support, or at unscheduled visits. Adverse events are managed according to grade and relatedness to study drug; closer monitoring may be recommended at any grade. Investigators are encouraged to modify or withhold study drugs possibly related to adverse events of grade 3 or higher. Additional guidance is provided in study standard operating procedures and by the CAC.

### Study endpoints {12}

#### Efficacy

The primary efficacy outcome is the proportion of participants with favourable outcome at week 73 (Table 4).

**Table 4:** Definition of primary treatment outcome {12}

Outcome definition	Definition of favourable outcome	Definition of unfavourable outcome
Proportion of participants with a favourable outcome at week 73	The outcome is not classified as unfavourable, and one of the following is true: 1. The last two culture results are negative. These two cultures must be taken from sputum samples collected on separate visits, the latest between weeks 65 and 73. 2. The last culture result (from a sputum sample collected between weeks 65 and 73) is negative, and either there is no other post-baseline culture result or the penultimate culture result is positive due to laboratory cross contamination, and bacteriological, radiological and clinical evolution is favourable. 3. There is no culture result from a sputum sample collected between weeks 65 and 73 or the result of that culture is positive due to laboratory cross contamination, and the most recent culture result is negative, and bacteriological, radiological, and clinical evolution is favourable.	Any of the following: 1. Replacement or addition of one or more investigational drugs in an experimental arm (failure). 2. Replacement or addition of two or more investigational drugs in the control arm (failure). 3. Initiation of a new MDR-TB treatment regimen after the end of the allocated study regimen and before week 73 (relapse). 4. Death from any cause. 5. At least one of the last two cultures, the latest being from a sputum sample collected between weeks 65 and 73, is positive in the absence of evidence of laboratory cross contamination (failure/relapse). 6. The last culture result (from a sputum sample collected between weeks 65 and 73) is negative; AND there is no other post-baseline culture result or the penultimate culture is positive due to laboratory cross contamination; and bacteriological, radiological, or clinical evolution is unfavourable (failure/relapse). 7. There is no culture result from a sputum sample collected between weeks 65 and 73 or it is positive due to laboratory cross contamination. AND the most recent culture is negative; and bacteriological, radiological or clinical evolution is unfavourable (failure/relapse); or the most recent culture result is positive in the absence of laboratory cross contamination. 8. The outcome is not assessable because there is no culture result from a sputum sample collected between weeks 65 and 73 or it is positive due to laboratory cross contamination. AND - There is no other post-baseline culture result or the most recent culture is positive due to laboratory cross contamination. - The most recent culture is negative and bacteriological, radiological, and clinical evolution is not assessable. 9. Previously classified as unfavourable in the present study (except for participants whose outcome at 39 weeks was unfavourable because it was unassessable).

Secondary efficacy outcomes are:

1. The proportion of participants with favourable outcome at week 39
2. The proportion of participants with favourable outcome at week 104
3. The proportion of patients who experienced failure or relapse at week 73 and at week 104
4. Early treatment response, which is assessed through the following: (a) proportion of patients with culture conversion at 8 weeks assessed in Mycobacteria Growth Indicator Tube (MGIT) culture method (and on Löwenstein-Jensen [LJ] culture medium where possible); (b) time to culture conversion assessed in MGIT system (and LJ where possible); and (c) change in time to positivity (TTP) in MGIT over 8 weeks

The definitions used for the primary efficacy outcome are shown in Table 4. Efficacy endpoints at weeks 39, 73, and 104 are validated by the CAC, the aforementioned committee of expert MDR-TB clinicians who do not provide any input on the study protocol and are not involved in the study analysis.

Although differences are not expected, the primary efficacy endpoints are also used to evaluate efficacy across linezolid-dose-reduction strategies.

#### Safety

The main safety outcomes are at 73 (and 104) weeks:

1. The proportion of patients who died of any cause
2. The proportion of participants with grade 3 or greater AEs, SAEs, and AESIs of any grade by 73 and 104 weeks (grading is determined using the MSF Severity Scale, which was derived from CTCAE v4.0, supplemented by DMID and DAIDS scales where necessary [44]. The following adverse events, at grade 3 or higher, are defined as AESI: “electrocardiogram QT corrected interval prolonged”; leukopenia, anaemia or thrombocytopenia; peripheral neuropathy; optic neuritis; and increase in alanine aminotransferase (ALT) or aspartate aminotransferase (AST).)

The endpoint for assessment of safety of the linezolid-dose-reduction strategies is severe linezolid-related toxicity, defined as grade 3 or higher linezolid-related AEs (leukopenia, anaemia, thrombocytopenia, peripheral neuropathy, and optic neuropathy), SAEs, and AEs requiring linezolid discontinuation.

### Analysis of the primary endpoint and analysis populations {20a}{20b}{20c}

The primary analysis will compare the proportions of participants with a favourable outcome at week 73 between each experimental arm and the control arm: for all pairwise comparisons, a two-sided 95% confidence interval of the difference will be estimated. The non-inferiority of an experimental arm compared to the control will be established if the difference in favourable outcome at week 73 is greater than the lower equivalence margin, i.e. if the lower bound of the one-sided 97.5% CI—which corresponds to the lower bound of the 2-sided 95% CI—is greater than or equal to -12%. We present two main reasons for the 12% non-inferiority margin. First, the 70% expected efficacy of the control regimen was already higher than that of the standard of care regimen, mitigating concerns about biocreep. Emerging data has suggested that the control arm, containing new and repurposed drugs, may be even more effective (e.g. 80%) [16, 45]. If so, any regimen established as non-inferior could be concluded, with 97.5% confidence, to have a favourable outcome frequency of at least 68%. Second, relative to the control arm and conventional regimens, the experimental regimens would result in a significantly reduced pill burden and treatment duration, expected improved tolerability, and increased adherence.

The family-wise type I error will be controlled by ordering the non-inferiority comparisons. A fixed sequence approach will be considered: the regimen with the highest proportion of favourable outcomes will be compared to the control. If non-inferiority is concluded, then a comparison between the control and the experimental regimen having the second highest proportion of favourable outcomes will be performed, and so on as long as non-inferiority is concluded. Once non-inferiority is not demonstrated, the comparisons will stop. All the previous comparisons will have been done at the full one-sided alpha level of 2.5%. Adjusted analyses on the primary endpoint will be also performed by controlling for covariates.

The main primary efficacy analyses will be performed on both modified intent-to-treat (mITT) and per-protocol (PP) populations for the non-inferiority comparisons. The mITT population contains all randomized participants with culture-positive, FQ-susceptible, and RR-TB. The PP population contains participants from the mITT population who completed the study without any major protocol deviation that impacts the assessment of the primary endpoint. Patients who discontinue the study for reasons other than death or loss to follow-up will be excluded from the PP. Sensitivity analyses will be performed in two other efficacy populations: the assessable population and the mITT plus those with baseline culture-negative tuberculosis.

Safety analyses will be performed on the safety population, which includes all enrolled participants who had received at least one dose of study treatment (as treated). A full description of the statistical methods, including handling of missing data and planned analyses, is detailed in the Statistical Analysis Plan.

### Sample size assumptions {14}

The sample size was estimated through simulations that considered (1) early efficacy responses (at weeks 8 and 39), (2) primary outcome (at week 73) with variations described below, (3) the expected number of non-inferior experimental arms, (4) the type I error (2.5% one-sided), (5) expected reductions in population from ITT to mITT (11%) and mITT to PP (10%), and (6) the non-inferiority margin (12%). Sample size estimates were calculated for 75% favourable outcome at 73 weeks in the non-inferior experimental arms. Favourable outcome frequency in the control arm was assumed to be 70% at 73 weeks. A sample size of 750 randomized participants provides power greater than 80% to demonstrate the non-inferiority of at least 1 (and up to 3) experimental regimens. The expected 75% 73-week response in the experimental arm reflects available data. This includes conventional-regimen treatment with delamanid that resulted in 74.5% favourable treatment outcome [20] and observational studies of short-course MDR-TB treatment that reported treatment success of greater than 80% [46–49]. The sample size simulations were performed in R (R Foundation for Statistical Computing, Vienna, Austria) using bootstrapping methods.

### Data collection, monitoring, and management {18a}{19}{21a}{22}{23}{27}

Data are collected and entered into an electronic case report form, in a web-based system that is compliant with International Conference on Harmonisation (ICH) Good Clinical Practice (GCP) guidelines. Range checks are programmed into the system. Designated study team members at each participating site perform real-time quality control and periodic quality assurance activities. Checks for consistency are implemented at the data entry level on site and centrally after data entry. Regular data review and data cleaning for quality control are organized in a blinded way and detailed in the Data Monitoring Plan. In addition, external monitoring is performed in accordance with the protocol specific requirements, ICH GCP guidance, and other applicable requirements.

Data are managed centrally by Epicentre. Additionally, safety data are also entered in a separate pharmacovigilance database at the centralized MSF Pharmacovigilance Unit. Appropriate medical and research records are maintained for the trial, in compliance with ICH E6 GCP and regulatory and institutional requirements. All study documents are coded with a study identification number. All study records are managed in a secure and confidential fashion.

All adverse events that occur during study are documented and followed to resolution or stabilization; in the case of AESIs and SAEs, this follow-up may extend beyond the normal study-reporting period. SAEs are notified, within 24 h of awareness, by the site principal investigator (or designee) to the MSF Pharmacovigilance Unit. All SAEs deemed related to one or more investigational product(s) and considered unexpected with the use of such products are reported to National Regulatory Authorities and national/local IRBs. All other SAEs are reported in an Annual Safety Report prepared by the MSF Pharmacovigilance Unit, and earlier if there are specific local regulations for more frequent reporting. In addition, safety data are reviewed semi-annually by an independent Data Safety Monitoring Board (DSMB), the members of which have expertise in clinical trials, MDR-TB, pharmacology, Bayesian adaptive randomization, and electrophysiology. Their functioning is detailed in the DSMB charter.

### Participant timeline

**Fig. 1:**
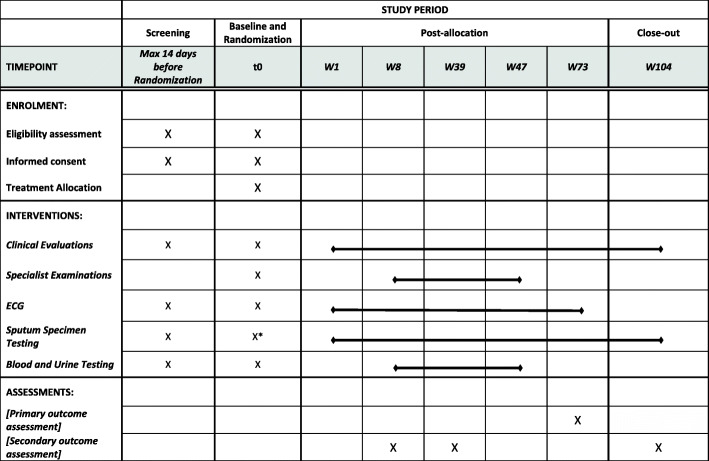
Schedule of enrolment, interventions, and assessments {12}{13}. *Sputum tests can be repeated at the baseline visit if needed for eligibility assessment

Figure 1 shows the schedule of eligibility assessment, visit procedures, and assessments over up to 104 weeks of study participation.

### Dissemination of trial findings {29}{31a}{31b}

The results of the trial will be disseminated under the responsibility of the principal investigators of the study. Investigators/study authors will have full access to the final trial dataset. Trial results will be published in peer-reviewed scientific journals and presented at national and international conferences, as appropriate. Results will be shared and discussed with study participants and affected communities. Authorship will be defined according to International Committee of Medical Journal Editors criteria. No professional writers will be involved.

### Biological specimens {26b}{33}

Subjects (and their legal representatives as applicable) are asked to provide written informed consent for storage and future use of *M. tuberculosis* isolates obtained from sputum samples. A subject may consent to study participation without consenting to future use of stored isolates. Stored isolates may be used only to improve diagnosis (including resistance testing) and treatment of TB. Relevant IRBs will oversee any future research using these specimens.

## Discussion

The lack of safe, effective, short, simple regimens for treatment of RR-/MDR-TB is a major challenge in TB control. Research shows that inadequately treated drug-resistant TB can undermine the population-level benefits of high rates of success in treatment of drug-susceptible TB [50]. Moreover, conventional, long, injectable-containing regimens have significant, negative quality-of-life impacts on patients with MDR-TB [51]. Shorter regimens have the potential to reduce loss to follow-up rates and treatment cost and can increase treatment access [52]. To maximize the potential impact of such shorter regimens on the existing gaps, the endTB trial uses an innovative approach. Instead of trying to select one single regimen—or an algorithm to develop individualized regimens—for all MDR-TB cases, endTB aims to identify multiple new regimens against FQ-susceptible TB as rapidly as possible. The trial assesses non-inferior (rather than superior) efficacy of the experimental regimens because of the significant benefits (detailed below) that could accrue to patients and health systems even in the absence of improved efficacy.

If safe and effective, endTB’s novel regimens have the potential to improve the current treatment options significantly. In the many settings that have not yet adopted all-oral, shorter regimens, endTB regimens could reduce treatment duration by more than 50%, eliminate injections and lessen the pill burden, and potentially increase efficacy without worsening toxicity. Some countries, including South Africa, have adopted routine use of shorter regimens in which bedaquiline has replaced the daily injectable [4, 53, 54]. This was based on encouraging results from observational research. To date, this evidence has been graded as low or very low certainty and WHO recommendations have been conditional for use of such regimens [4]. Relevant for settings that have hesitated, as well as for those that have adopted these innovations, the endTB trial will provide high-quality, direct evidence about specific regimens compared to the current standard of care.

The endTB trial complements other efforts, recently completed, planned or ongoing, to optimize treatment for drug-resistant TB. In addition, a pharmacokinetic substudy will inform optimized dosing and offer insights into pharmacokinetic and pharmacodynamic drug-drug interactions. endTB focuses on questions, populations, or regimens and/or provides additional (high-quality) evidence compared to other studies. For example, Opti-Q (NCT01918397) optimized the dose of levofloxacin [55]; when available, efficacy findings from that study could be incorporated into use of any levofloxacin-containing endTB regimens found to be non-inferior to the current standard of care. The endTB observational study expanded the use of novel compounds (bedaquiline and delamanid) and repurposed older agents (linezolid and clofazimine) that had limited prior population exposure for TB. Delivered within longer and sometimes injectable-sparing regimens, preliminary results are encouraging [56]. Shorter regimens are the subject of other studies. STREAM 1 established the non-inferiority of the shorter “Bangladesh” regimen to an internal control that did not include treatment innovations that were occurring concurrently with the trial (e.g. use of bedaquiline, delamanid, or linezolid). And, toxicity of the STREAM regimen was found to be significant [16]. STREAM II (NCT02409290) adds bedaquiline to regimens (longer and shorter) containing drugs that have been used historically for DR-TB.

A more recent development, the SRA licensure of pretomanid was the first marketing authorization for a novel agent (in the same nitroimidazole class as delamanid) used within a new, shorter regimen. Prospects are promising for this regimen containing pretomanid, bedaquiline, and linezolid (BPaL); currently WHO recommends its use only under research conditions [4]. Additional research is required in light of certain characteristics of the pivotal study: the small sample size (*N*=109); lack of an internal, concurrent control; single-country setting; and the high rate of toxicity with the dose of linezolid examined [57–60]. The uncontrolled ZeNix trial, expected to report in 2021, aims to optimize linezolid dosing within the BPaL regimen (NCT03086486) in a more heterogeneous patient population. Lastly, TB-PRACTECAL (NCT02589782) assesses shorter regimens containing bedaquiline and pretomanid; its phase 3 component examines the BPaL regimen supplemented by moxifloxacin. It has recently been stopped early based on promising, unpublished interim results in the experimental arm. endTB prioritizes the combination of bedaquiline and delamanid, in order to maximize the information available to clinicians and policymakers. endTB’s implementation in a highly heterogeneous MDR-TB patient population—in seven countries in Africa, Eastern Europe, Asia, and the Americas—will result in evidence that is generalizable to most MDR-TB patients, including HIV-positive patients and adolescents. Such a strong evidence base is most informative for global policy and has been shown to lead to better country uptake [61]. This would result in wider-spread adoption of injectable-sparing, shorter regimens that likely improve patient compliance and simplify the logistical burden on health care systems. These changes can also make treatment accessible to a much higher proportion of patients with drug-resistant TB.

Like Opti-Q, STREAM, STREAM II, and TB-PRACTECAL, endTB employs the gold standard of the randomized, concurrently, controlled trial. Several other design decisions were made to “future-proof” the trial despite the established lengthy time required to enrol trial participants for DR-TB [62]. These decisions maximize efficiency and will facilitate endTB’s relevance at the time results become available. First, compared to sequential trials, the inclusion of five concurrent experimental arms reduces substantially the sample size and time required to evaluate each of the experimental arms. Second, deployment of Bayesian response-adaptive randomization further reduces the sample size (and time required) by approximately 20% compared to fixed randomization [33]. Limitations of this approach include the resources required, reliance on an unvalidated interim endpoint to inform adaptation, and possible bias introduced by changes in the study population over time. These limitations have been discussed elsewhere [33]. Third, endTB has allowed the control regimen to keep pace with changing global (and local) policy guidance, protecting its relevance at the end of the study. endTB was among the first trials to allow the use of bedaquiline and delamanid in the comparator. While TB-PRACTECAL also includes this innovation, STREAM did not. When WHO endorsed for the first time the shorter regimen for use in a subset of patients with DR-TB [63], endTB amended the protocol and other study documentation to permit the use of the WHO-endorsed shorter regimen in the control arm. Similarly, the WHO recommendation to remove the injectable from conventional (and shorter) regimens [4] was also adopted for endTB control arm regimens. In the Nix-TB trial, the study included no concurrent control [57]. Instead, outcomes in the cohort receiving the BPaL regimen were compared to those in a historical cohort. The comparator excluded patients who received drugs included in the Nix regimen despite their common inclusion in standard-of-care treatment contemporaneous to the Nix trial [64]. In the case of the STREAM 1 trial, although non-inferiority was established, it was in comparison to standard of care that had become obsolete during the trial. In the endTB trial, the planned comparison to an evolving standard of care does complicate interpretation relative to an ideal situation in which all control-arm participants received the standard of care in force at trial’s end. For the sample sizes and length of follow-up currently required for phase 3 trials of drug-resistant TB treatment, and the current dynamic nature of treatment recommendations, this ideal is unlikely to be realized. However, endTB’s approach permits the possibility of post hoc comparisons between experimental arms and different compositions of control arms and enhances the chances of meaningful results at the time of study completion. Fourth, endTB is also powered for the improved treatment response expected with recent changes to the standard of care as evidenced by the control arms in the STREAM 1 and delamanid phase 3 trials [45]. endTB sample size simulations accounted for a smaller expected difference between experimental and control arms than that anticipated in the delamanid phase 3 trial. The surprisingly small difference between treatment responses in the control and experimental arms may have been a factor in the “negative” results of the pivotal trial of delamanid [45]. Although a modest improvement in treatment response would not have been considered clinically meaningful in a comparison of two longer, injectable-containing regimens, such a result in one or more shorter, all-oral endTB regimens could be transformative. Fifth, the “hybrid” follow-up approach adopted by endTB balances the objectives of producing results as rapidly as possible while ensuring the ability to detect the majority of relapse cases in the experimental arms. This approach, supported by a re-analysis of relapse timing in trials of shorter TB therapy, was first proposed by Nunn and colleagues [65] as a way to dramatically reduce time to study results with minimal impact on relapse detection. In the endTB trial all participants assigned to experimental arms will have more than 8 months of post-treatment follow-up and 90% are expected to have more than 12 months of post-treatment observation.

endTB results will not inform treatment of FQ-resistant TB. endTB ensures the use of FQ in patients who may be able to benefit from it. And, the endTB consortium has embarked on a second, complementary clinical trial, “Evaluating newly approved drugs in combination regimens for multidrug-resistant tuberculosis with fluoroquinolone resistance” (endTB-Q; NCT03896685), examining fluoroquinolone-sparing regimens only in patients who are not likely to benefit from that drug class. endTB-Q represents, to date, the only randomized, concurrently controlled trial with adequate power to detect treatment effects in patients with fluoroquinolone-resistant TB.

Change in policy and practice around treatment of drug-resistant TB has historically been slow. In the absence of randomized, internally, and concurrently controlled trials, the urgent need for improved treatments, however, has understandably driven recent acceptance of evidence from observational and uncontrolled studies. The endTB trial will close a critical gap by providing high-quality evidence around the safety and efficacy of five injectable-sparing, 9-month multidrug regimens for the treatment of MDR/RR TB.

## Trial status

endTB recruitment began in the first site in February 2017; with expansion to additional sites, enrolment is expected to be complete by mid-2021.

## Data Availability

Not applicable.

## Abbreviations

DSMB: Data Safety Monitoring Board
GCP: Good Clinical Practice
HMS: Harvard Medical School
ICH: International Conference on Harmonisation
IRB: Institutional Review Board
IRD: Interactive Research & Development
ITM: Institute of Tropical Medicine
MDR-TB: Multidrug-resistant tuberculosis
mITT: Modified intent-to-treat
MSF: Médecins Sans Frontières
PIH: Partners In Health
PP: Per protocol
RR: Rifampin-resistant
TB: Tuberculosis
XDR: Extensively drug-resistant
